# Effects of Frailty on Outcomes Following Surgery Among Patients With Hip Fractures: A Systematic Review and Meta-Analysis

**DOI:** 10.3389/fmed.2022.829762

**Published:** 2022-03-23

**Authors:** Yan Ma, Ansu Wang, Yijiao Lou, Daojuan Peng, Zhongyan Jiang, Tongxia Xia

**Affiliations:** ^1^Department of Orthopaedic Surgery, Affiliated Hospital of Zunyi Medical University, Zunyi, China; ^2^Nursing School, Zunyi Medical University, Zunyi, China; ^3^Department of Nursing, Affiliated Hospital of Zunyi Medical University, Zunyi, China

**Keywords:** frailty, hip fractures, mortality, postoperative complications, meta-analysis

## Abstract

**Background:**

Frailty is a syndrome of multisystem dysfunction in the elderly. The association between preoperative frailty and postoperative outcomes in patients with hip fractures is unclear. To address this issue, we performed a meta-analysis to determine the association of frailty with postoperative mortality, complications, and readmission in patients with hip fractures.

**Methods:**

We searched PubMed, Web of Science, Embase, and The Cochrane Library for cohort studies of frailty associated with postoperative adverse events in patients with hip fractures from inception to November 6, 2021. The Newcastle-Ottawa Scale was used to evaluate the quality of the included literature. Statistical analysis of meta-analysis was performed using Review Manager 5.3.

**Results:**

Twelve retrospective cohort studies and seven prospective cohort studies involving a total of 62,132 patients met the inclusion criteria for this meta-analysis. Compared with non-frail patients, the pooled results showed that frailty was associated with patient in-hospital mortality (relative risk [RR] = 2.93; 95% confidence intervals [CI]: 2.56–3.34), 30-day mortality (RR = 2.85, 95%CI: 1.67–4.85) and total complications (RR = 1.79, 95%CI: 1.50–2.15). Subgroup analysis showed that the type of study design and frailty assessment tool had no significant effect on the results. Sensitivity analysis showed that the polled results of frailty predicted one-year mortality and 30-day readmission was unstable.

**Conclusions:**

In this meta-analysis, we found that preoperative frailty may be associated with postoperative adverse events in patients with hip fractures, including in-hospital mortality, 30-day mortality, and postoperative complications.

**Systematic Review Registration:** PROSPERO, identifier: CRD42021287739.

## Introduction

Hip fractures are a serious public health problem, mainly in the elderly (1). It is estimated that the number of hip fractures worldwide will be 4.5 million by 2050 (2). In Italy, the number of elderly people with hip fractures has increased by 28.62 percent in 15 years (3). Hip fracture severely affects patients' postoperative life and has a high mortality (1, 4). Mortality was 13.3% during hospitalization or within 30 days, and 8.4–36% within 1 year after surgery (5, 6). Surgical treatment is the main treatment for hip fracture patients, but there is a high incidence of postoperative complications and readmission (7). It is necessary to adopt effective methods to assess the risk of postoperative death or adverse events and to classify patients according to the risk.

Frailty is a symptom of old age in which the functions of multiple physiological systems decline, and the incidence of frailty increases with age (8, 9). Identification of frailty is the premise of intervention and management. Early identification and intervention of frailty can reduce the adverse consequences of frailty. For the screening and diagnosis of frailty in the elderly, there is a lack of exact laboratory indicators, and various scales are commonly used for evaluation (10). The contents of various frailty assessment scales mainly include physiological, psychological, social and environmental aspects. Fried Frailty Phenotype, Frailty Index, and FRAIL are commonly used assessment tools (11–13). Compared with the commonly used preoperative risk assessment tools, frailty assessment scales are more specific to the disease characteristics of the elderly (14). Frailty patients often have clinical manifestations such as falls and delirium, so frailty is often associated with poor prognostic function and mortality. Several studies have shown that frailty is associated with postoperative mortality and adverse events in patients with hip fractures (15, 16). However, individual studies have come to different conclusions (14). There was few systematic review of the relationship between frailty and hip fracture outcomes.

Therefore, we performed a meta-analysis of the value of preoperative frailty in predicting postoperative outcomes of hip fractures, including mortality, readmission, and complications.

## Methods

The study was conducted based on the Meta-analysis Of Observational Studies Epidemiology (MOOSE) guidelines (17) and has been registered on PROSPERO.

### Search Strategy

Two authors conducted a literature search independently, until November 6, 2021. Databases include PubMed, Web of Science, Embase, and The Cochrane Library. The search terms include “hip fractures” OR “femoral neck fracture” OR “Trochanteric Fracture” OR “Intertrochanteric Fracture” OR “Subtrochanteric Fracture” AND “frailty” OR “frail.” In addition, we manually searched the reference list of the included literature to ensure possible inclusion. Any inconsistencies will be discussed and resolved with the third author.

### Inclusion and Exclusion Criteria

The inclusion criteria of this meta-analysis were as follows: (i) the study was a cohort study; (ii) patients with hip fractures in adults (age > 18 years); (iii) patients were assessed for frailty; (iv) studies must report at least one of the following outcomes: mortality or postoperative complications or 30 days readmission. The following types of studies will be excluded: (i) reviews, letters, editorial articles, conference abstracts, or editorial articles; (ii) duplicate literatures.

### Data Extraction

Two authors independently extracted information from the included literature using standard Excel spreadsheets and checked by a third author. The extracted information includes: author, year, country, age, study design, sample, assessment tool and outcomes. Mortality was the main outcome, including in-hospital mortality, 30-day mortality, 1-year mortality. Secondary outcomes were 30-day readmission, postoperative delirium, pneumonia and total complications.

### Quality Assessment

The Newcastle-Ottawa Scale (NOS) was used to evaluate the quality of the included cohort studies (18). The NOS is a nine-point scale with three domains. Articles with NOS scores above seven are considered to be of high quality. Two authors independently assessed the quality of the included studies using NOS and checked by a third author.

### Statistical Analysis

All statistical analyses were performed using Review Manager 5.3 (The Cochrane Collaboration, Oxford, UK). In this meta-analysis, we calculated relative risk (RR) and 95% confidence intervals (CIs) for mortality, 30-day readmission, and postoperative complications. We performed a subgroup analysis of study types and assessment tools. Heterogeneity between studies was assessed by *I*^2^ statistic and Chi-square test. When *I*^2^ ≥ 50% (*p* < 0.05), significant heterogeneity between studies was considered, and the fixed-effects model was used. In addition, we use the random-effects model. Sensitivity analysis was performed by deleting each study separately. In this meta-analysis, an unadjusted *P* < 0.05 was considered statistically significant.

## Results

### Study Selection

**Figure 1:**
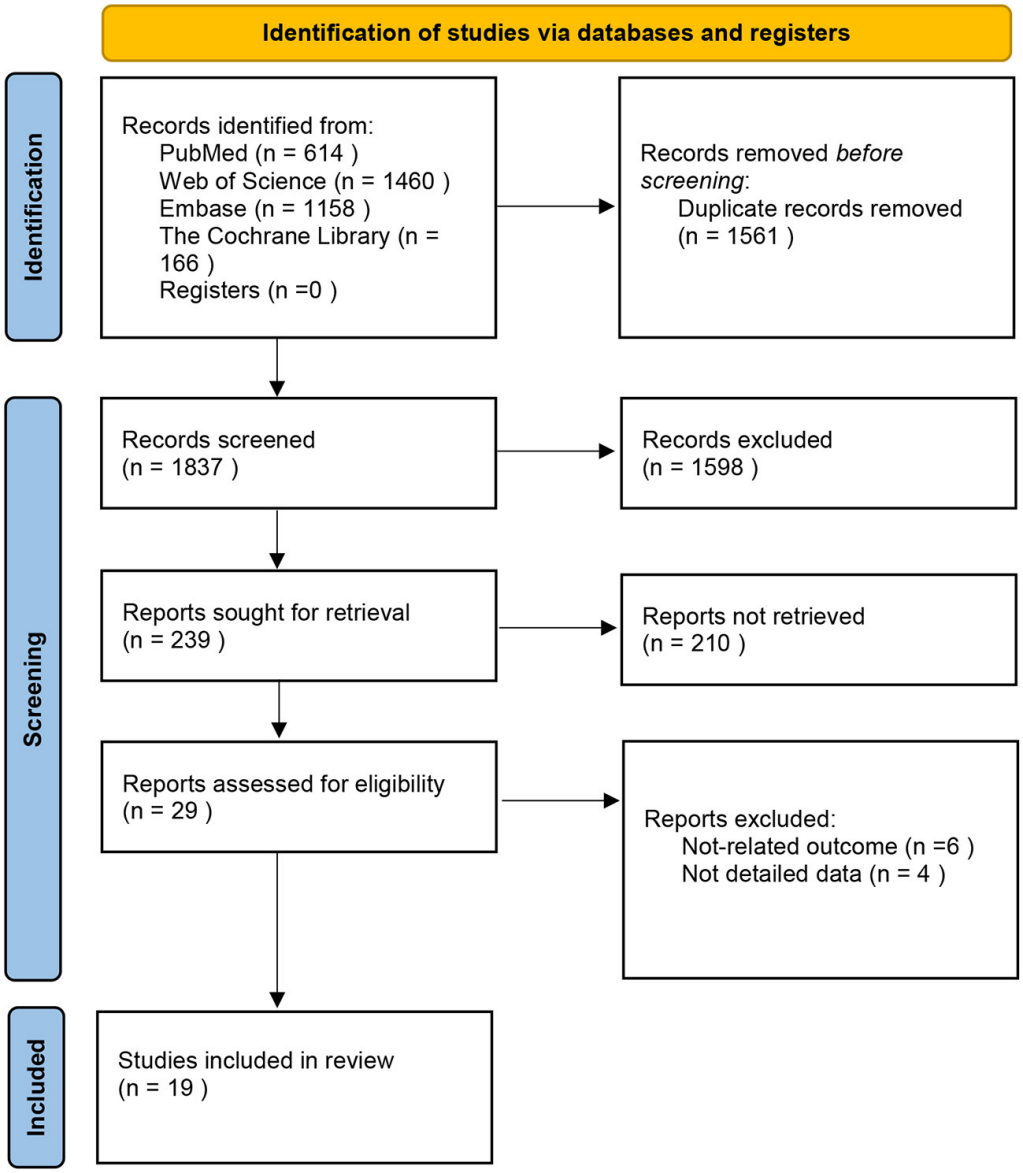
Preferred reporting items for systematic reviews and meta-analyses flow diagram (19).

Figure 1 shows the literature screening flow chart of this study. We initially retrieved 3,398 articles, and after removing duplicates, there were 2,076 articles. After reading the titles and abstracts, 1,598 were excluded. After reading the original texts according to the pre-specified criteria, 19 studies were included in this review (14–16, 20–34).

### Study Characteristics

Table 1 shows the main characteristics of the included studies in this meta-analysis. Seven prospective cohort studies and 12 retrospective cohort studies were published between 2014 and 2021. A total of 62,132 patients were assessed for frailty in 19 studies, of which 16,789 were assessed as frailty. Elderly female patients were the main population, accounting for about 61%. Mortality was the primary outcome measure in this meta-analysis, which was reported in 13 studies. Twelve studies reported secondary measures of the study. Of the included studies, five were from the US, three were from China, two each from the UK, Italy and Australia, and one each from Canada, the Netherlands, Singapore, Germany and Japan. All studies scored between 6 and 9 on the NOS assessment, indicating that the included studies were medium or high quality (Supplementary File).

**Table 1:** Characteristics of included studies.

**Author**	**Year**	**Country**	**Age^a^**	**Study Design**	**Sample**	**Female**	**Frailty**	**Definition of frailty**	**Outcomes^b^**
Chan	2019	Canada	82.5 ± 8.4	retrospective	423	267	279	Clinical Frailty Scale	1, 5, 6, 7
Chen	2019	Taiwan, China	53–97	prospective	245	166	55	Chinese-Canadian Study of Health and Aging Clinical Frailty Scale	2, 4
Gandossi	2021	Italy	-	prospective	988	250	360	Frail Index	6
Gleason	2017	USA	82.3 ± 7.4	retrospective	175	131	73	FRAIL Scale	2, 4, 5, 6, 7
Jorissen	2020	Australia	85.8 ± 6.3	retrospective	4,771	3,607	1,307	Frail Index	2, 3
Kistler	2015	USA	86 ± 4	prospective	35	29	18	Modified Fried Frailty Index	5, 6, 7
Krishnan	2014	UK	81	prospective	178	131	122	Frail Index	1, 2
Kua	2016	Singapore	79.1 ± 9.6	retrospective	82	55	28	Reported Edmonton Deal Scale	5, 6, 7
Meyer	2020	Germany	65.0 ± 11.4	retrospective	4,558	2,477	210	Hospital Frailty Risk Score	6, 7
Narula	2020	Australia	82.7 ± 9.1	retrospective	509	374	338	Clinical Frailty Scale	1, 2, 3
Patel	2014	USA	81.1 ± 8.5	retrospective	481	-	200	Modified Frail Index	3
Pizzonia	2021	Italy	86.6 ± 5.9	prospective	364	284	264	modified 19-item Frailty Index	3
Shen	2021	China	76.7 ± 8.8	retrospective	965	637	126	Modified Frail Index	5, 7
Shimizu	2021	Japan	83.6 ± 6.7	retrospective	31,692	19,707	11,431	Hospital Frailty Risk Score	1, 4, 6, 7
Shin	2016	USA	65.2	retrospective	14,584	8,109	861	Modified Frail Index	2, 5
Thorne	2021	UK	-	retrospective	1,299	-	822	Clinical Frailty Scale	1, 2
Wilson	2019	USA	73.7 ± 12.7	retrospective	377	192	117	Modified Frail Index	1, 5
Winters	2018	Netherlands	83.0 ± 6.6	prospective	286	215	160	Veiligheids Management Systeem	2
zhu	2020	China	78.9 ± 6.5	prospective	120	55	18	FRAIL Scale	5, 6, 7

a *Mean or range*.

b *1. inpatient mortality, 2. 30-day mortality, 3. 1-year mortality, 4. 30-day readmission, 5. total complications, 6. postoperative delirium, 7. Pneumonia*.

### Mortality

**Figure 2:**
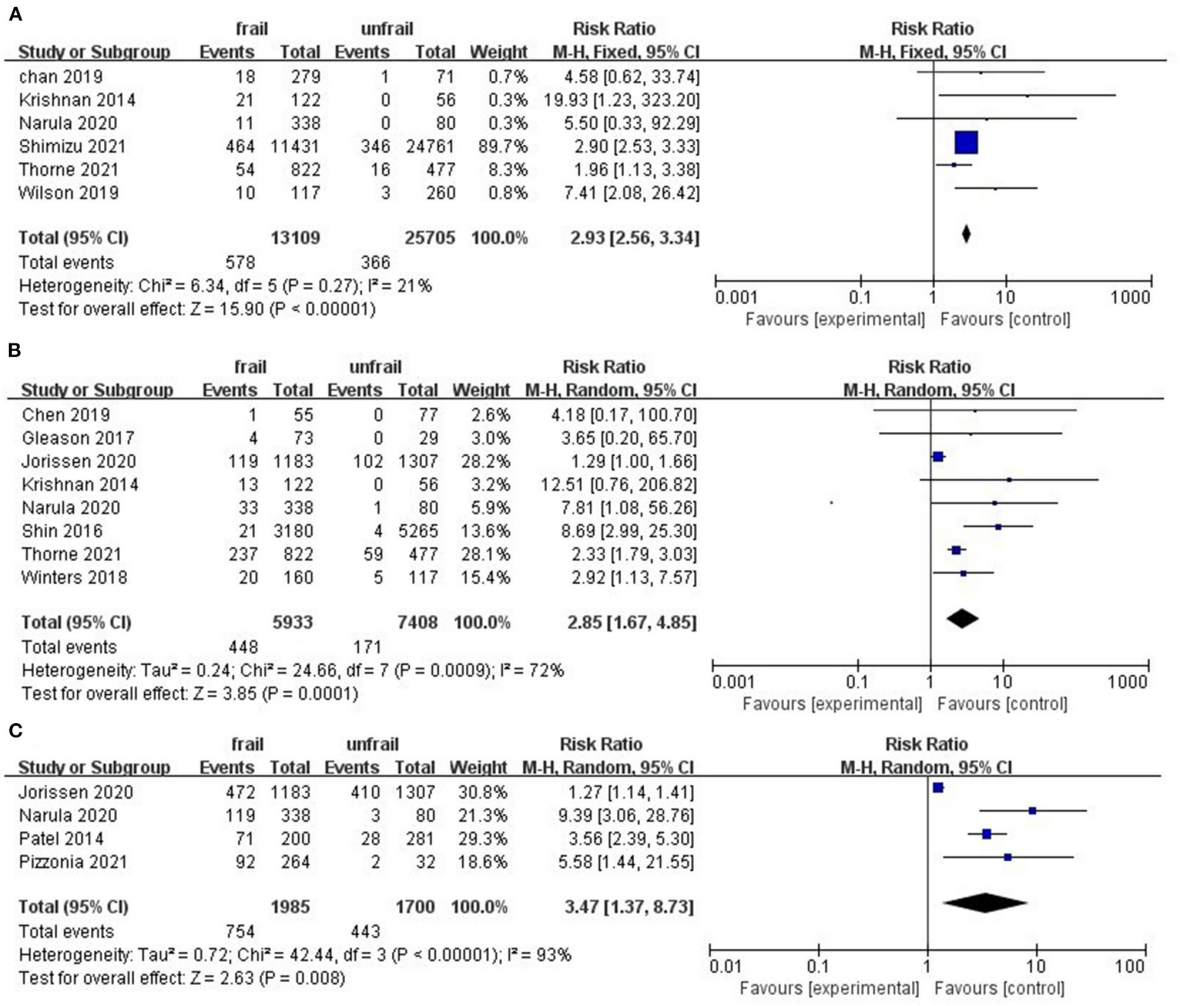
Forest plots of frailty and postoperative mortality of hip fractures. **(A)** in-hospital mortality **(B)** 30-day mortality **(C)** one-year mortality.

Among the included studies, 13 studies involving 55,384 hip patients reported the association between frailty and mortality. We extracted in-hospital mortality from 6 studies, 30-day mortality from 8 studies, and one-year mortality from 4 studies. The pooled results using fixed-effect models showed that frailty was associated with in-hospital mortality (RR = 2.93; 95%CI: 2.56–3.34; I^2^ = 21%; Figure 2A). Similar results were obtained by sensitivity analysis of the 30-day mortality pooled results (RR: 2.88–3.13). Due to there was greater heterogeneity between 30-day mortality studies (RR = 2.85, 95%CI: 1.67–4.85; I^2^ = 72%; Figure 2B) and one-year mortality studies (RR = 3.42, 95%CI: 1.30–9.01; I^2^ = 94%; Figure 2C), random-effects models were used for both groups. Sensitivity analysis showed that Jorissen's study (22) was the main source of heterogeneity. When this study was removed, heterogeneity was reduced in 30-day mortality (RR = 3.58, 95%CI: 2.11–6.09; I^2^ = 29%) studies and the results still showed a strong predictive relationship between frailty and mortality. However, sensitivity analysis of the pooled results of 1 year mortality showed that the results were not stable. Subgroup analysis showed that study design type and assessment tool had no significant effect on results (Table 2).

**Table 2:** Subgroup analysis of frailty associated with mortality in patients with hip fracture.

**Group standard**		**Inpatient mortality**				**30-Day mortality**			
		***N***	**RR (95%CI)**	**I^**2**^**	**P (heterogeneity)**	***N***	**RR (95%CI)**	**I^**2**^**	**P (heterogeneity)**
Study design type	Retrospective	5	2.96 [2.59, 3.40]	8	0.36	5	2.68 [1.44, 4.98]	83	0.0003
	Prospective	1	23.94 [1.42, 402.64]	Not applicable	Not applicable	3	3.45 [1.45, 8.20]	0	0.6
Frailty assessment tool	CFS	3	2.33 [1.37, 3.98]	0	0.61	2	2.86 [1.16, 7.05]	31	0.23
	Other	3	3.01 [2.63, 3.45]	22	0.27	6	3.42 [1.33, 8.83]	71	0.004

### Complications and 30-Day Readmission

**Figure 3:**
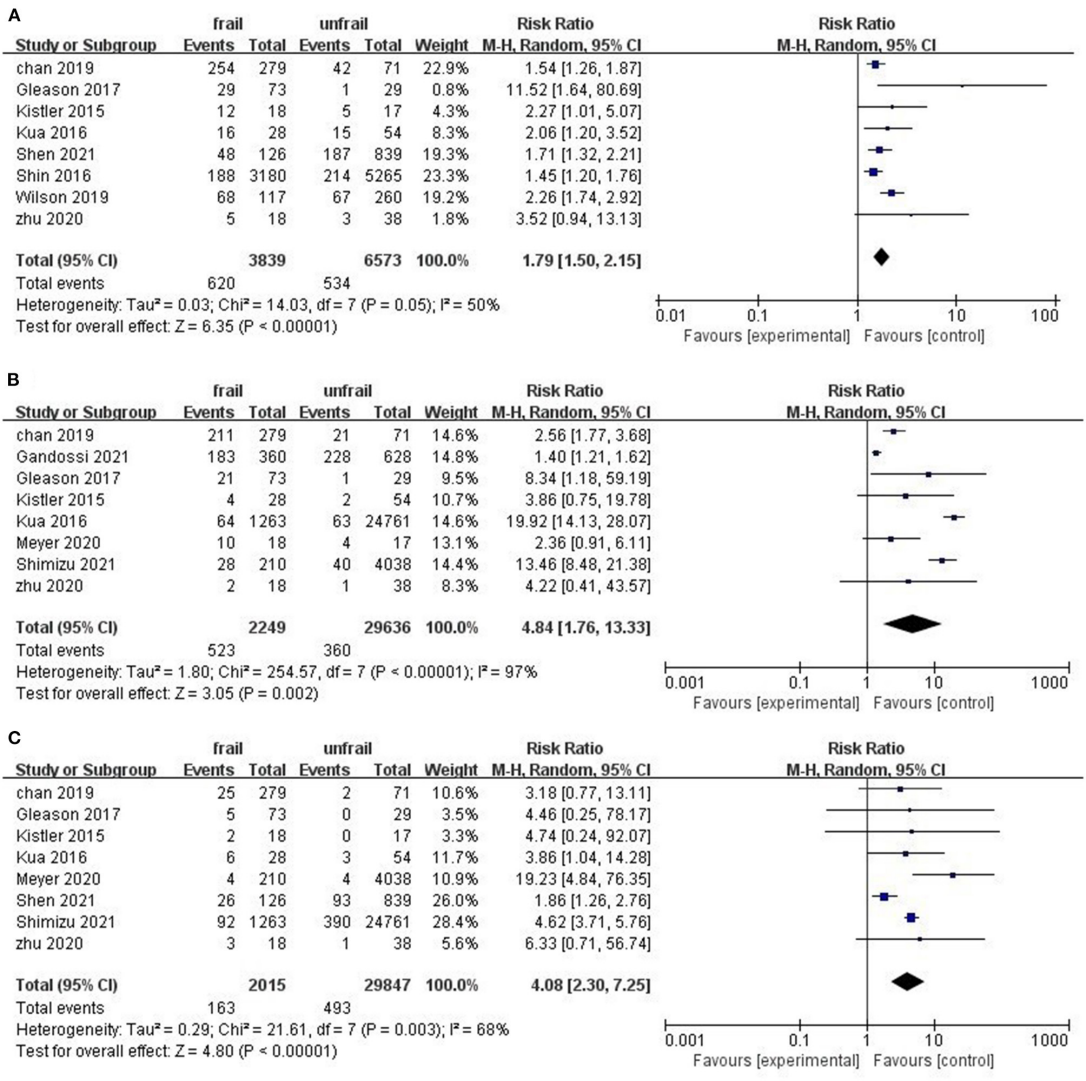
Forest plots of frailty and postoperative complications of hip fractures. **(A)** Total complications **(B)** postoperative delirium **(C)** pneumonia.

**Figure 4:**
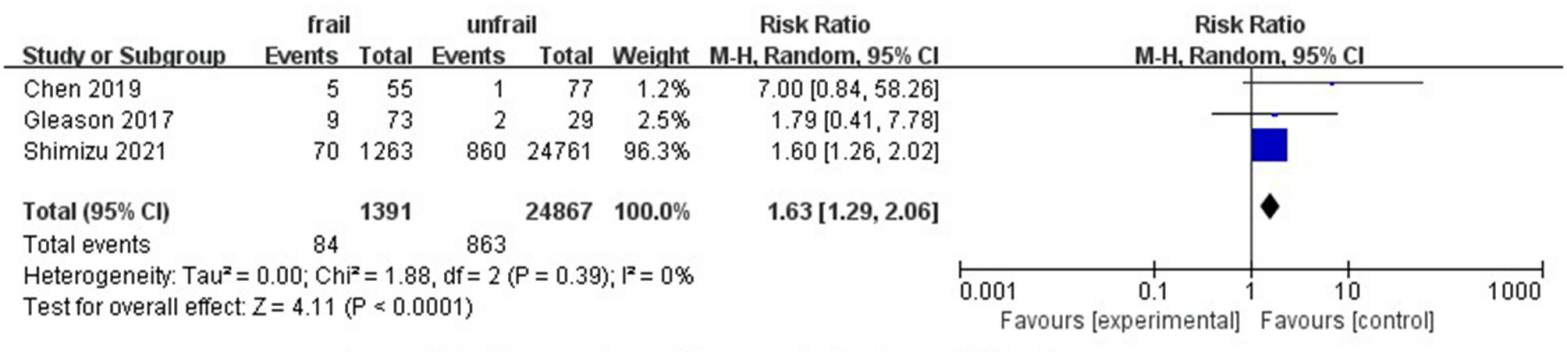
Forest plots of frailty and 30-day readmission of hip fractures.

Eight studies involving 10,476 patients reported total postoperative complications. The pooled results indicate that preoperative frailty has a significant impact on postoperative complications (RR = 1.79, 95%CI: 1.50–2.15; I^2^ = 50%; Figure 3A). Sensitivity analysis showed that the results were reliable. Postoperative delirium and pneumonia were the most frequently reported complications (Figures 3B,C). Only three studies noted a relationship between frailty and hospital readmission in patients with hip fractures. Due to low heterogeneity between studies, we used the fixed effects model. Results of the meta-analysis showed that frailty was associated with patient readmission (RR = 1.63, 95%CI: 1.29–2.06; I^2^ = 0%; Figure 4). Because one of the studies had too much weight in the meta-analysis, we conducted a sensitivity analysis. When the study was removed, frailty and 30-day readmission were no longer statistically significant.

## Discussion

To our knowledge, this study is the first systematic review and meta-analysis of frailty predicting outcomes in patients with hip fractures. The pooled results showed that preoperative frailty was significantly associated with postoperative mortality and complications in patients with hip fractures compared with no-frail patients.

Frailty, one of the most common syndromes in the elderly, refers to a decrease in physical reserves and the body's ability to resist stress (35). Frailty is associated with musculoskeletal disease. Loss of muscle and bone mass in the elderly can lead to sarcopenia and osteoporosis, especially in older postmenopausal women (36). Hip fractures are a serious complication of osteoporosis and are usually treated surgically. The mortality rate is one of the most important indicators of hip surgery due to severe trauma and poor prognosis (37). This meta-analysis showed that preoperative frailty was associated with postoperative hospital mortality and 30-day mortality, independent of study type and frailty assessment tools. The results are consistent with studies of patients with other conditions that require surgery, including lung cancer and digestive tumors (38, 39). However, the association between frailty and one-year mortality is uncertain, this may be due to the different selection of confounding factors in different studies. More studies are needed to determine the validity of frailty as a predictor of long-term mortality after hip fracture surgery.

Although frailty increases postoperative mortality, surgery is still necessary for patients with hip fractures. In order to increase the survival rate of patients with preoperative frailty, corresponding medical and nursing measures should be formulated (40). Accurate frailty assessment is a prerequisite for effective intervention. A variety of different scales were used to assess frailty in the studies included in this meta-analysis, but these scales do not include the characteristics of patients with hip fractures. It is uncertain which scale is more appropriate for patients with hip fractures. Fried Criteria is one of the most widely used assessment tools, but in comparing Modified Fried Criteria to Reported Edmonton Deal Scale (REFS) to predict clinical outcomes in patients with hip fractures, REFS showed even greater advantages (11, 16). The prevalence of frailty varied widely in this study, although the results of the subgroup analysis showed that the assessment tools did not affect frailty in predicting mortality. Although frailty and fractures are most common in older people, they can also occur in younger people. In addition to the assessment tools, differences in patient age may account for the larger difference in frailty. Future research should develop frailty assessment tools for patients with hip fractures to more accurately assess patients and increase comparability between studies.

Postoperative complications seriously affect the quality of life of patients, and even directly lead to the death of patients. Pneumonia, delirium, deep vein thrombosis and infection are common postoperative complications in patients with hip fracture (41). Our meta-analysis showed that frailty was an effective predictor of total postoperative complications. However, there are inevitably differences in the types of total complications involved. For example, delirium was not considered in Shen's study because evaluation of delirium required an in-person visit and postoperative delirium was not recorded in the patient's medical records. Postoperative delirium is characterized by acute disturbance of consciousness with altered cognitive function. Our study showed that frailty significantly increased the risk of postoperative delirium, but there was too much heterogeneity between studies and we did not find a cause for the heterogeneity. Of the studies on frailty predicting postoperative complications, only one was a prospective cohort and the sample size was very small. Therefore, prospective studies with large sample sizes should be conducted in the future to further determine the impact of frailty on postoperative complications of hip fractures.

The reliability of preoperative frailty as a predictor of 30-day readmission is uncertain. Only three studies reported 30-day readmission outcomes, and two of them were not statistically significant. Readmission rate is an important indicator of medical quality and the Hospital Readmission Reduction Program is used to punish high readmission rate (42). Early postoperative readmission of patients with hip fracture not only increases the risk of death, but also increases the economic burden and social medical burden of patients (43). Patients may be admitted to different medical facilities within 30 days of discharge, and this information is difficult to obtain. It is necessary to strengthen the communication between medical institutions on patient readmission information.

There are some limitations in this meta-analysis that need to be considered. Firstly, due to different statistical methods and confounding variables selected in each study, unadjusted data were adopted for meta-analysis. This may increase the heterogeneity of the study and thus affect the validity of the effect values. Second, due to the lack of detailed data, we were unable to conduct subgroup analyses of specific fracture sites. Third, multiple frailty assessment tools were used in the included studies, which led to inevitable heterogeneity. And several studies have blurred the line between frailty and non-frailty. Finally, the studies included in our study are all cohort studies and several studies included small sample sizes. Due to the limitations of observational studies, there may be potential bias in this study.

## Conclusion

In conclusion, preoperative frailty may significantly affect the outcome of patients with hip fractures, including in-hospital mortality, 30-day mortality, and postoperative complications. However, more research is needed to confirm the impact of frailty on long-term mortality and readmission. In addition, future research should focus on developing frailty assessment tools specifically for hip fractures.

## Data Availability Statement

The original contributions presented in the study are included in the article/supplementary material, further inquiries can be directed to the corresponding author.

## Author Contributions

YM, AW, and TX were responsible for the conception and design of the study, drafting and revising the manuscript. YM, YL, and DP conducted literature retrieval, screening, and quality evaluation. YM and ZJ analyzed the data and explained the results. All authors contributed to the article and approved the submitted version.

## Funding

This study was supported by Zunyi City Science and Technology Project (No. HZ[2021]142) and Guizhou Province Science and Technology Project (No. LC[2021]038).

## Conflict of Interest

The authors declare that the research was conducted in the absence of any commercial or financial relationships that could be construed as a potential conflict of interest.

## Publisher's Note

All claims expressed in this article are solely those of the authors and do not necessarily represent those of their affiliated organizations, or those of the publisher, the editors and the reviewers. Any product that may be evaluated in this article, or claim that may be made by its manufacturer, is not guaranteed or endorsed by the publisher.
