# Supplementary material for: Effects of Frailty on Outcomes Following Surgery Among Patients With Hip Fractures: A Systematic Review and Meta-Analysis
Source: Front Med (Lausanne). 2022 Mar 23;9:829762. doi: 10.3389/fmed.2022.829762 (PMC8984086; doi:10.3389/fmed.2022.829762)
Supplement: Supplementary file 1 [file Table_1.docx]

| study | Selection | | | | Comparability | | Outcome | | | Total |
| --- | --- | --- | --- | --- | --- | --- | --- | --- | --- | --- |
|  | 1 | 2 | 3 | 4 | 1a | 1b | 1 | 2 | 3 |  |
| chan 2019 | 1 | 1 | 1 | 1 | 0 | 1 | 1 | 1 | 1 | 8 |
| Chen 2019 | 1 | 1 | 1 | 1 | 1 | 1 | 1 | 1 | 1 | 9 |
| Gandossi 2021 | 1 | 1 | 1 | 1 | 0 | 1 | 1 | 1 | 1 | 8 |
| Gleason 2017 | 1 | 1 | 1 | 1 | 0 | 1 | 1 | 1 | 1 | 8 |
| Jorissen 2020 | 1 | 1 | 1 | 1 | 0 | 1 | 1 | 1 | 1 | 8 |
| Kistler 2015 | 1 | 1 | 1 | 1 | 0 | 0 | 1 | 1 | 1 | 7 |
| Krishnan 2014 | 1 | 1 | 1 | 1 | 0 | 1 | 1 | 1 | 1 | 8 |
| Kua 2016 | 1 | 1 | 1 | 1 | 0 | 0 | 1 | 1 | 1 | 7 |
| Meyer 2020 | 1 | 1 | 1 | 1 | 0 | 1 | 1 | 1 | 1 | 8 |
| Narula 2020 | 1 | 1 | 1 | 1 | 0 | 0 | 1 | 1 | 1 | 7 |
| Patel 2014 | 1 | 1 | 1 | 1 | 0 | 0 | 1 | 1 | 1 | 7 |
| Pizzonia 2021 | 1 | 1 | 1 | 1 | 0 | 0 | 1 | 1 | 1 | 7 |
| Shen 2021 | 1 | 1 | 1 | 1 | 0 | 1 | 1 | 1 | 1 | 8 |
| Shimizu 2021 | 1 | 1 | 1 | 1 | 1 | 1 | 1 | 1 | 1 | 9 |
| shin 2016 | 1 | 1 | 1 | 1 | 0 | 1 | 1 | 1 | 1 | 8 |
| Thorne 2021 | 1 | 1 | 1 | 1 | 0 | 0 | 1 | 1 | 1 | 7 |
| Wilson 2019 | 1 | 1 | 1 | 1 | 0 | 0 | 1 | 1 | 1 | 7 |
| Winters 2018 | 1 | 1 | 1 | 1 | 0 | 0 | 1 | 1 | 1 | 7 |
| zhu 2020 | 1 | 1 | 1 | 1 | 0 | 0 | 1 | 1 | 1 | 7 |

**Quality assessment of included studies by the Newcastle–Ottawa Scale**
